# Out-of-Band Response for the Coastal Zone Imager (CZI) Onboard China’s Ocean Color Satellite HY-1C: Effect on the Observation Just above the Sea Surface

**DOI:** 10.3390/s18093067

**Published:** 2018-09-12

**Authors:** Tingwei Cui, Jing Ding, Fujuan Jia, Bing Mu, Rongjie Liu, Pengmei Xu, Jianqiang Liu, Jie Zhang

**Affiliations:** 1 First Institute of Oceanography, State Oceanic Administration, Qingdao 266061, China; liurj@fio.org.cn (R.L.); zhangjie@fio.org.cn (J.Z.); 2 National Satellite Ocean Application Service, Beijing 100081, China; dingjing@mail.nsoas.org.cn (J.D.); jqliu@mail.nsoas.org.cn (J.L.); 3 Beijing Institute of Space Mechanics & Electricity, Beijing 100190, China; jiafujuan_new@163.com (F.J.); 13651065845@163.com (P.X.); 4 Department of Physics, Ocean University of China, Qingdao 266071, China; mubing@ouc.edu.cn

**Keywords:** out-of-band response, Coastal Zone Imager (CZI), HY-1

## Abstract

The out-of-band (OOB) response is one of the key specifications for satellite optical sensors, which has important influences on quantitative remote sensing retrieval. In this paper, the effect of OOB response on the radiometric measurements made just above the sea surface is evaluated for the three broad visible bands (i.e., blue, green, and red) of the Coastal Zone Imager (CZI) onboard China’s ocean satellite HY-1C to be launched in September 2018. For the turbid coastal (Case 2) waters whose optical properties are mainly dominated by suspended sediment and colored dissolved organic material, the OOB effect can be neglected (<2%) for all three CZI visible bands. For the phytoplankton-dominated (Case 1) waters which are mainly distributed in the clear open ocean, a significant (>2%) OOB effect was found in the green band over oligotrophic waters (chlorophyll a concentration ≤~0.1 mg/m^3^), and accordingly a model based on the CZI blue-green band ratio is proposed to correct this effect. The OOB influence on the CZI ocean color retrieval is discussed. This research highlights the importance of the comprehensive pre-launch radiometric characterization and the OOB effect correction for the broad band space-borne sensor, in order to achieve a high-quality quantitative ocean product.

## 1. Introduction

The international ocean satellite family will soon have two new members, HY-1C in 2018 and HY-1D in 2019, which will constitute China’s optical satellite constellation for global ocean monitoring (one in the morning and the other in the afternoon). The two satellites are equipped with the same optical instruments. Except the Chinese Ocean Color and Temperature Scanner (COCTS) for the ocean color and sea surface temperature monitoring of the global ocean, another key payload is the Coastal Zone Imager (CZI), which can provide four broad band (i.e., blue, green, red, and near infrared) optical images with a wide swath of 950 km and moderate spatial resolution (50 m), for the coastal environment and resources monitoring from space. The wide swath is achieved by mosaicking the images acquired by the two identical cameras, each having a 32° field of view, with 1° overlapping.

Through qualitative image interpretation, CZI will aid coastal zone management by providing key information on reclamation [1], wetlands [2], land cover/use [3], mangroves [4], coral reef [5], and sea ice [6] in the coastal zone and islands, where significant changes are usually expected due to intense human activity. At the same time, the quantitative information inversion from CZI images may also be expected to retrieve key parameters of aquatic environments (including turbidity, suspended sediment concentration, transparency, and water depth) [7,8,9], natural resources (such as wetland biomass) [10], and abnormal ecological phenomena [11,12]. In order to achieve this goal, the comprehensive optical characterization of CZI is of high importance, including the radiometric accuracy, signal-to-noise ratio, out-of-band (OOB) response, etc. 

For the multi-spectral sensor, especially those with wide band width, the sensor-observed signal includes not only the contribution from sensor’s in-band response, but also that from the unexpected OOB response [13]. Typically, the in-band radiometric contribution is defined as the integration of the input signal over the spectral domain within 1% peak response, whereas the OOB radiometric contribution is the difference between the in-band contribution and the total radiometric contribution, defined as the input signal integrated over the whole spectral domain [14]. 

Significant OOB response can lead to the shifting of the actual center wavelength relative to the nominal one (defined as the center of the 50% peak response), and the OOB-induced radiometric bias will have negative impact on the satellite-derived product [14]. Thus, the comprehensive prelaunch OOB assessment (and correction) is of vital importance for high-accuracy satellite data processing and high-quality products [13]. 

As the OOB radiometric contribution is determined not only by the sensor’s spectral response, but also by the observed target radiance, a systematic OOB effect evaluation and correction should be performed for different types of waters [14]. Besides, as the top-of-the atmosphere (TOA) radiance (or reflectance) measured by the satellite sensor is dominated by the atmosphere radiometric contribution, the result of OOB evaluation based on this signal will mainly reflect the OOB modulation on the atmosphere signal, whereas the OOB influence on the ocean radiometric observation is less effectively characterized. 

In this background, the CZI OOB characteristics will be evaluated for turbid (Case 2) and clear (Case 1) waters [15], both of which will be covered in the CZI global observation of the world’s coastal zones and islands. In order to effectively characterize the sensor’s OOB effect on the in-water ocean color retrieval, the OOB analysis is based on the sensor-observed signal just above the sea surface (i.e., the spectral remote sensing reflectance R_rs_(*λ*)), rather than the TOA radiance, due to the fact that all the ocean biological and biogeochemical parameters are derived from R_rs_(*λ*). The evaluation results are expected to benefit the quantitative parameter retrieval.

## 2. Materials and Method 

**Figure 1:**
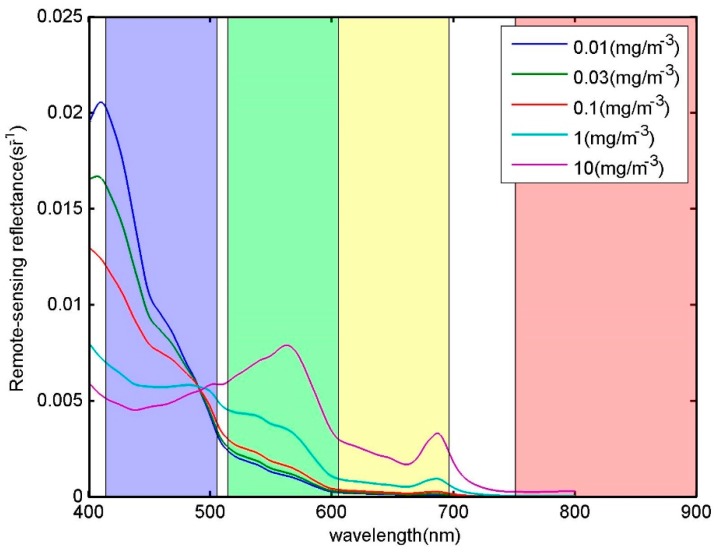
Spectral remote sensing reflectance R_rs_(*λ*) of Case 1 water, simulated by Hydrolight software under various chlorophyll a concentrations. The overlaid colored areas denote the four bands of CZI (Coastal Zone Imager), whose boundaries are determined by the 1% peak response.

For the Case 1 water, whose optical properties are dominated by phytoplankton, the R_rs_(*λ*) in the range of 350~800 nm was simulated using the Hydrolight radiative transfer software [16] with the embedded bio-optical model for Case 1 water [17,18]. As a numerical forward model, Hydrolight is characterized by its clear physics and faster computation speed, and has been widely used and extensively validated. In the Case 1 bio-optical model, the absorption from pure seawater, phytoplankton, and colored dissolved organic matter, as well as backscattering from pure seawater and phytoplankton was considered. The input chlorophyll a concentrations (Chla) for simulation were 0.01 mg/m^3^, 0.03 mg/m^3^, 0.1 mg/m^3^, 1 mg/m^3^, and 10 mg/m^3^. The simulated R_rs_(*λ*) is shown in Figure 1. 

**Figure 2:**
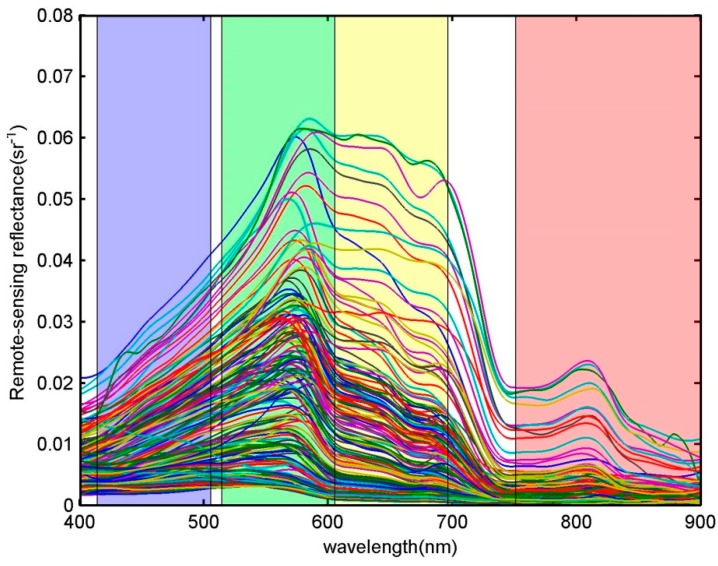
Spectral remote sensing reflectance R_rs_(*λ*) of Case 2 water, measured from turbid coastal waters along the east China coast (*n* = 748). The overlaid colored areas denote the four CZI bands, whose boundaries are determined by the 1% peak response. The lines of different colors represent the data measured at different stations.

For the Case 2 water, R_rs_(*λ*) data were acquired from observations in turbid waters along the east China coast (*n* = 748), as shown in Figure 2. The suspended particulate material (SPM) and colored dissolve organic matter (CDOM), rather than phytoplankton, were the dominant ocean color components [19,20,21]. For our in situ data, the SPM concentration ranged from 0.10~1762.13 mg/L with an average of 28.30 ± 103.85 mg/L. The ranges of CDOM and chlorophyll a concentrations were 0.09~0.42 m^−1^ (0.18 ± 0.10 m^−1^) and 2.03 ± 2.22 mg/m^3^ (0.06~14.99 mg/m^3^), respectively.

**Figure 3:**
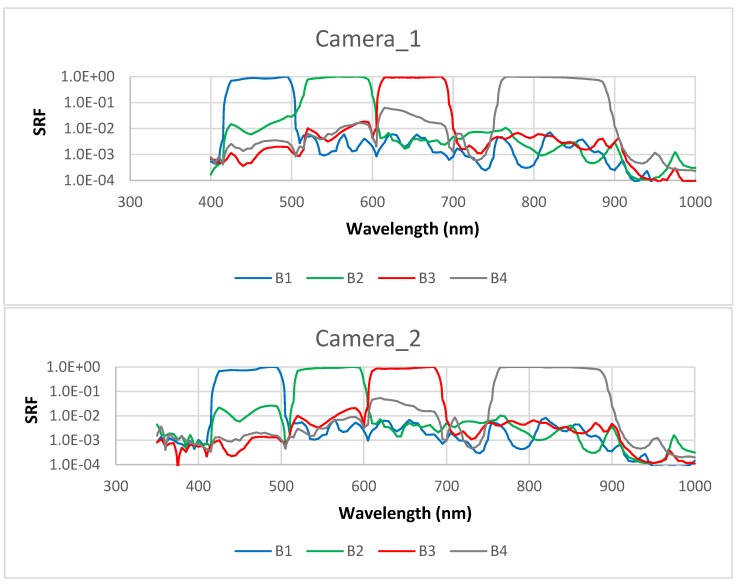
Spectral response functions (SRF, in arb. units) of the two cameras of CZI. The CZI’s wide swath of 950 km is achieved by mosaicking the images acquired by the two cameras, each having 32° field of view, with 1° overlapping.

For the *i*-th band *λ_i_*, OOB(Δ) (difference) and OOB(%) (relative difference) is calculated as follows [14].

(1) $$\mathrm{OOB}\left(\lambda_{i}\right)\left(\Delta \right)\;=\;R_{rs}^{\left(Total\right)}\left(\lambda_{i}\right)-R_{rs}^{\left(In-Band\right)}\left(\lambda_{i}\right)\;$$

where $R_{rs}^{\left(Total\right)}\left(\lambda_{i}\right)$ and $R_{rs}^{\left(In-Band\right)}\left(\lambda_{i}\right)$ are the observed total and in-band signals for band *λ_i_*.

(2) $$R_{rs}^{\left(Total\right)}\left(\lambda_{i}\right)\;=\;\frac{\int_{All}^{}R_{rs}\left(\lambda \right)F_{0}\left(\lambda \right)S_{i}\left(\lambda \right)d\lambda }{\int_{All}^{}F_{0}\left(\lambda \right)S_{i}\left(\lambda \right)d\lambda }\;$$

(3) $$R_{rs}^{\left(In-Band\right)}\left(\lambda_{i}\right)\;=\;\frac{\int_{S_{i}\left(\lambda^{\left(\pm \right)}\right)\;=\;1\%}^{}R_{rs}\left(\lambda \right)F_{0}\left(\lambda \right)S_{i}\left(\lambda \right)d\lambda }{\int_{S_{i}\left(\lambda^{\left(\pm \right)}\right)\;=\;1\%}^{}F_{0}\left(\lambda \right)S_{i}\left(\lambda \right)d\lambda }\;$$

where $F_{0}\left(\lambda \right)$ is extra-terrestrial incident solar irradiance [22] and $S_{i}\left(\lambda \right)$ is the spectral response function (SRF) for the *i*-th CZI band (shown in Figure 3). For the in-band response calculation, the integration is performed over the spectral domain around the peak response from $\lambda^{\left(-\right)}$ corresponding to $S_{i}\left(\lambda^{\left(-\right)}\right)\;=\;1\%$ to $\lambda^{\left(+\right)}$ corresponding to $S_{i}\left(\lambda^{\left(+\right)}\right)\;=\;1\%$ [14]. 

From $\mathrm{OOB}\left(\lambda_{i}\right)\left(\Delta \right)$, the relative out-of-band difference $\mathrm{OOB}\left(\lambda_{i}\right)\left(\%\right)$ can be derived.

(4) $$\mathrm{OOB}\left(\lambda_{i}\right)\left(\%\right)\;=\;\frac{\mathrm{OOB}\left(\lambda_{i}\right)\left(\Delta \right)}{R_{rs}^{\left(In-Band\right)}\left(\lambda_{i}\right)}\times 100\;$$

According to the definition given by Reference [14], the OOB correction factor is the ratio of the signal at the nominal center wavelength of each band, $R_{rs}^{}\left(\lambda_{i}^{\left(N\right)}\right)$, to the actual signal observed by the corresponding band $R_{rs}^{\left(Total\right)}\left(\lambda_{i}\right)$, as shown in Equation (5). The closer the correction factor is to 1.0, the smaller the OOB effect.

(5) $$\mathrm{Corr}\left(\lambda_{i}\right)\;=\;\frac{R_{rs}^{}\left(\lambda_{i}^{\left(N\right)}\right)}{R_{rs}^{\left(Total\right)}\left(\lambda_{i}\right)}\times 100\;$$

After $\mathrm{OOB}\left(\lambda_{i}\right)\left(\Delta \right)$, $\mathrm{OOB}\left(\lambda_{i}\right)\left(\%\right)$, and $\mathrm{Corr}\left(\lambda_{i}\right)$ were derived for each station in turbid waters, these parameters were averaged for each band. The above calculations were confined to the blue, green, and red bands due to the fact that the signal in the CZI near infrared band (750–905 nm) is quite small and can usually be neglected, except the highly turbid waters with a high load of suspended sediment.

From the spectral response function shown in Figure 3, the nominal central wavelength *λ_N_*, which is defined as the center of the two wavelengths with 50% peak response, was determined for the four bands of the two CZI cameras (Table 1). Also shown in this table is the in-band spectral domain bounded with 1% spectral response around the peak response. Through comparison of the specifications of the two cameras, it can be found that for the blue (B1), red (B3), and near infrared (B4) bands, the two cameras share almost the same nominal central wavelengths, and the spectral interval with 50% (1%) peak response. The obvious difference between the two cameras was only found in the green band (B2), in terms of the wavelength with 1% response shorter than the peak response (464 nm vs. 511 nm, shown as bold numbers in Table 1), and thus the band interval (144 nm vs. 97 nm).

As the significant OOB contribution will lead to the variability of the measured $R_{rs}^{}\left(\lambda \right)$ at the nominal center wavelength, the effective band center wavelength for the *i*-th band λ_i_, $\lambda_{i}^{\left(E\right)}$, is determined using Equation (6), and then the central wavelength shift (∆*λ* = *λ_E_* − *λ_N_*_)_ is quantified accordingly [14].

(6) $$R_{rs}^{}\left(\lambda_{i}^{\left(E\right)}\right)\;=\;R_{rs}^{\left(Total\right)}\left(\lambda_{i}\right)\;$$

## 3. OOB Effect Evaluation

The result for the Case 1 water is shown in Table 2. Generally speaking, the larger OOB values (≥2%, shown as bold numbers) were mainly found in the oligotrophic waters (with low Chla) and at longer bands, where the water-leaving signals were weak; a clear decreasing trend of OOB for the three bands exists with elevated Chla (except the green band in eutrophic waters). 

Specifically, among the three visible bands, the blue band showed the lowest OOB, with no more than −1% for both cameras, and the OOB effect can be neglected accordingly, at least for the concerned Chla range of 0.01 mg/m^3^~10 mg/m^3^. The OOB in the red band ranged from ~2% to ~20% for both cameras, which resulted from the low level of water-leaving radiance in the clear waters. It was also noted that in the blue and red bands, the two CZI cameras had almost the identical OOB response characteristics. 

**Figure 4:**
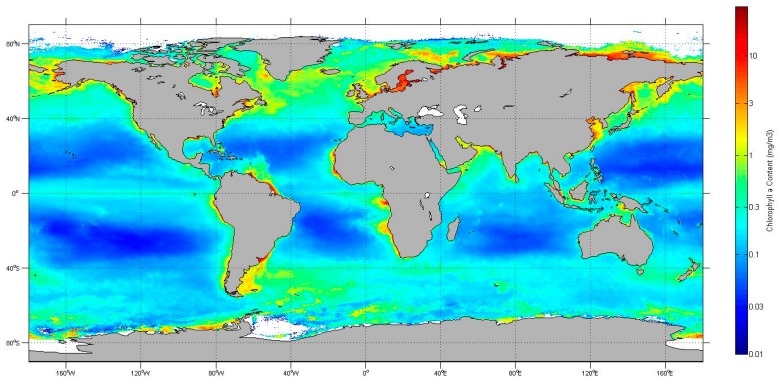
Annual average of the global chlorophyll a concentration distribution in 2010, which was merged from AQUA MODIS (Moderate-resolution imaging spectra-radiometer), ENVISAT MERIS (Medium-spectral Resolution Imaging Spectrometer), and FY-3 MERSI (Medium Resolution Spectral Imager) [23]. The areas in dark blue are characterized by low Chla (≤0.1 mg/m^3^), where the noticeable OOB (out-of-band) effect (≥2%) in the CZI green band need be corrected.

For the green band, the OOB of the two cameras showed some differences, which were in the range of −0.9%~4.6% and −1.1%~12.8%, respectively. Their major discrepancy (4%~8%) was found in the oligotrophic waters (with Chla of 0.01 mg/m^3^~0.1 mg/m^3^). In addition, on the whole for the waters with Chla less than ~0.1 mg/m^3^, the OOB effect cannot be neglected, and needs to be accounted for properly. As an example, Figure 4 shows the satellite derived global Chla distribution [23] from which it is clear that the waters with Chla ≤ 0.1 mg/m^3^ were mainly located in the sub-tropic gyres of the Pacific, Atlantic, and Indian Oceans, far from the coast which is the hotspot of CZI observation.

The central wavelength shifts of the blue, green, and red bands for Camera 1 (Camera 2) are in the range of −4.5~13.5 nm (−2.5~13.5 nm), −8.5~20.5 nm (−9.5~19.5 nm), −20~−24 nm (−18.5~−21.5 nm), respectively, with Chla ranging from 0.01 mg/m^3^ to 10 mg/m^3^. 

Table 3 lists the OOB results for the Case 2 water. The largest OOB, which is less than ~2%, is found in the red band and can be neglected. The averages of central wavelength shifts in the blue, green and red bands for the Camera 1 (Camera 2) are 2.4 nm (3.1 nm), −13.7 nm (−13.7 nm), and −15.5 nm (−13.9 nm), respectively. 

## 4. OOB Effect Correction

**Figure 5:**
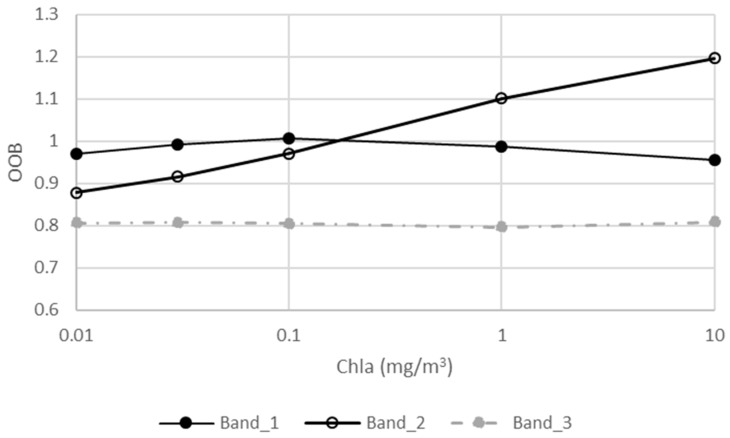
OOB correction factors for the three CZI visible bands in Case 1 water with chlorophyll a concentrations from 0.01 mg/m^3^ to 10 mg/m^3^. The two CZI cameras had almost the same correction factors (relative difference <2.8%), so results from only one camera is shown here.

For Case 1 water (Table 2 and Figure 5), two cameras had consistent OOB correction factors, with a percentage difference of less than 2.8%. Specifically, the correction factors for the blue band were the closest to 1.0 (0.956~1.007) among the three CZI visible bands, implying the most insignificant OOB effect, which is consistent with the OOB evaluation results described above. Not surprisingly, the values for the red band were found to be the lowest (0.786~0.809), indicating the most significant OOB effect, due to the weak water-leaving signal at this band. The values of correction factors for the green band have the largest variation magnitude (0.879~1.197), and a close correlation between the correction factor and Chla (R^2^ = 0.99) was found. 

**Figure 6:**
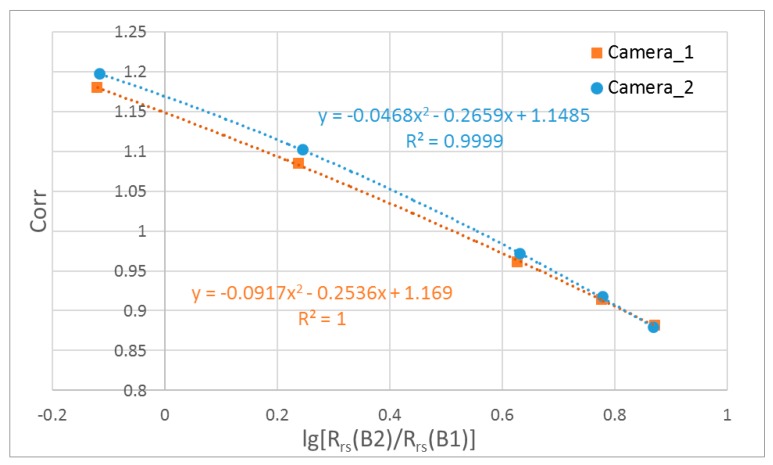
Estimation of the OOB correction factor for the CZI green band in Case 1 water from band ratios of CZI remote sensing reflectance.

As Chla can be remotely sensed from the blue-to-green band ratios of CZI R_rs_(*λ*), the OOB correction factor for the green band can be estimated from the ocean color data itself (Figure 6), as shown in Equations (7) and (8). The results for the two cameras (Equation (7) vs. Equation (8)) have a slight difference (~2%).

(7) $$\mathrm{OOB}\left(B2\_\_Camera\_1\right)\;=\;-0.0468\;X^{2}-\;0.2659X\;+\;1.1485\;$$

(8) $$\mathrm{OOB}\left(B2\_Camera\_2\right)\;=\;-0.0917X^{2}\;-\;0.2536X\;+\;1.1690\;$$

$$X\;=\;\log_{10}\left[\frac{R_{rs}\left(B2\right)}{R_{rs}\left(B1\right)}\right]\;$$

For the turbid waters, the OOB correction factors (Table 3) for the CZI blue, green, and red bands ranged from 0.962 to 1.099, and the two cameras also had consistent results.

## 5. Discussion

The evaluation and correction of the CZI OOB effect in this paper was performed by adopting the approach proposed by Reference [14], whose effort was dedicated for the visible bands of major satellite ocean color sensors of SeaWiFS (Sea-Viewing Wide Field-of-View Sensor), MODIS, and MERIS) characterized by a narrow band width. They found that for turbid coastal and inland waters, OOB responses of the above three sensors were insignificant, which is consistent with our CZI results for turbid Case 2 waters. For the Case 1 waters, they found obvious OOB response for SeaWiFS in 555 nm, VIIRS in 551 nm, and MODIS in 412 nm, whereas our results indicate significant OOB response in the CZI green band. The differences between our and their results can be attributed to the spectral response function characteristics of various sensors. It is also noted that in order to effectively evaluate the OOB effect on the ocean optical signal and in-water retrieval, our OOB analysis was based on the physical quantity of remote sensing reflectance, which was the sensor-observed signal just above the sea surface without atmosphere interference (as Reference [14] did), rather than the top-of-atmosphere (TOA) radiance which is measured at the satellite altitude and dominated by the atmosphere contribution. 

The sensor’s OOB response may have had a negative impact on the quantitative ocean color retrieval, which needs careful consideration. For example, for the Chla retrieval, which is usually based on the blue-green band ratio algorithm [24], the algorithm accuracy was proportional to the difference between the uncertainties of the remote sensing reflectance in the blue and green bands [25]. In addition, a 3% OOB response would lead to the positive bias in the derived Chla for the Case 1 water [26]. Our results indicate that the CZI green band had significant OOB response in the oligotrophic Case 1 water, and thus the OOB correction was highly recommended for the quantitative ocean color retrieval, especially for the Chla estimation. 

Different from the dedicated ocean color sensors for open ocean monitoring, which are characterized by multiple bands, narrow band width (typically 10~20 nm), and low spatial resolution (~1 km), the CZI onboard HY-1C was designed for coastal zone monitoring with a major focus on land monitoring. This kind of sensor is characterized by the limited number of bands, wide band width, and relatively high spatial resolution (compared with ocean color data). Importantly, in the near future we will see more similar sensors in orbit. In 2019, satellite HY-1D will be launched, carrying the same CZI as that onboard HY-1C. After 2020, HY-1E and HY-1F satellites will send another two CZIs into space, whose spatial resolution will be increased (from 50 m) to ~5 m. From these satellite images, quantitative ocean information with fine spatial structure (at meter scale) can be expected. In order to achieve this goal, the comprehensive prelaunch optical characterization, especially the OOB evaluation, is highly important for developing quantitative ocean products, as demonstrated here.

## 6. Conclusions

Based on the sensor-observed signals just above the sea surface, the OOB response was evaluated for the three visible bands (i.e., blue, green, and red) of the two CZI sensors onboard the HY-1C satellite, which will be launched in September 2018. The evaluation in the Case 1 (clear) and Case 2 (turbid) waters shows that the OOB response of the two sensors was consistent. For the phytoplankton-dominated (Case 1) water, the significant (>2%) OOB effect was found in the green band over the oligotrophic waters (Chla ≤ ~0.1 mg/m^3^), which is far from the coast which is the hotspot of CZI observation. The OOB effect can be corrected by a proposed model based on the CZI blue-green band ratio of spectral remote sensing reflectance. For the turbid coastal (Case 2) waters dominated by suspended sediment and colored dissolved organic material, the OOB effect can be neglected (≤~2%) for the three visible bands. This research highlights the importance of comprehensive pre-launch radiometric characterization and the OOB evaluation for the space-borne sensor designed for coastal zone monitoring, especially for quantitative ocean information retrieval.

## Figures and Tables

**Table 1:** Band specifications for the two cameras of HY-1C CZI. The nominal center wavelengths (defined as the center of the 50% peak response) and band intervals with 50% (1%) peak response are determined from the spectral response functions. The specifications for the two cameras are found to be almost identical with exceptions in the green band, shown as bold numbers.

		Nominal Central Wavelength/nm	Band Interval with 50% Response/nm	Band Interval with 1% Response/nm
**Camera 1**	B1	461.5	423–500 (77)	415–505 (90)
	B2	557.5	518–597 (79)	**464**–608(**144**)
	B3	651.0	610–692 (82)	604–700 (96)
	B4	823.0	759–887(128)	750–902(152)
**Camera 2**	B1	461.5	423–500 (77)	415–505 (90)
	B2	558.0	518–598 (80)	**511**–608 (**97**)
	B3	649.5	609–690 (81)	602–700 (98)
	B4	825.5	761–890(129)	750–905(155)

**Table 2:** The OOB and correction factor for the three visible bands (i.e., blue-B1, green-B2, and red-B3) of the two CZI cameras in the Case 1 water. OOB higher than 2% is highlighted as bold numbers. *λ_N_* and *λ_E_* denote the nominal center wavelength and the effective center wavelength, respectively. ∆*λ* represents the shift of the effective central wavelength *λ_E_* relative to a nominal one *λ_N_*.

		Camera 1						Camera 2					
Chla (mg/m^3^)		*λ_N_* (nm)	*λ_E_* (nm)	∆*λ* (nm)	OOB (Δ)	OOB (%)	Correction factor	*λ_N_* (nm)	*λ_E_* (nm)	∆*λ* (nm)	OOB (Δ)	OOB (%)	Correction Factor
0.01	B1	461.5	457.0	−4.5	−7.50 × 10^−5^	−0.8	0.9566	461.5	459.0	−2.5	−9.10 × 10^−5^	−0.9	0.9701
	B2	557.5	549.0	−8.5	5.83 × 10^−5^	**4.6**	0.8818	558.5	549.0	−9.5	1.51 × 10^−^^4^	**12.8**	0.8786
	B3	651.0	631.0	−20.0	2.67 × 10^−5^	**19.1**	0.7855	649.5	631.0	−18.5	2.50 × 10^−5^	**17.7**	0.8069
0.03	B1	461.5	459.0	−2.5	−6.60 × 10^−5^	−0.8	0.9804	461.5	461.0	−0.5	−8.00 × 10^−5^	−0.9	0.9923
	B2	557.5	550.0	−7.5	4.38 × 10^−5^	**3.1**	0.9138	558.5	551.0	−7.5	1.26 × 10^−^^4^	**9.5**	0.9166
	B3	651.0	631.0	−20.0	2.59 × 10^−5^	**14.7**	0.7904	649.5	631.0	−18.5	2.46 × 10^−5^	**13.9**	0.8081
0.1	B1	461.5	461.0	−0.5	−5.50 × 10^−5^	−0.7	0.9980	461.5	463.0	1.5	−6.70 × 10^−5^	−0.9	1.0066
	B2	557.5	554.0	−3.5	2.60 × 10^−5^	1.5	0.9611	558.5	556.0	−2.5	9.56 × 10^−5^	**5.8**	0.9707
	B3	651.0	630.0	−21.0	2.60 × 10^−5^	**10.1**	0.7912	649.5	630.0	−19.5	2.53 × 10^−5^	**9.8**	0.8048
1	B1	461.5	475.0	13.5	−3.30 × 10^−5^	−0.6	0.9860	461.5	475.0	13.5	−4.10 × 10^−5^	−0.7	0.9869
	B2	557.5	567.0	9.5	−1.20 × 10^−5^	−0.4	1.0841	558.5	568.0	9.5	2.38 × 10^−5^	0.7	1.1011
	B3	651.0	627.0	−24.0	3.27 × 10^−5^	**4.5**	0.7871	649.5	627.0	−22.5	3.43 × 10^−5^	**4.7**	0.7967
10	B1	461.5	472.0	10.5	−9.70 × 10^−^^6^	−0.2	0.9599	461.5	472.0	10.5	−1.20 × 10^−5^	−0.2	0.9563
	B2	557.5	578.0	20.5	−5.70 × 10^−5^	−0.9	1.1791	558.5	578.0	19.5	−7.30 × 10^−5^	−1.1	1.1965
	B3	651.0	628.0	−23.0	4.28 × 10^−5^	1.8	0.7973	649.5	628.0	−21.5	4.76 × 10^−5^	2.0	0.8088

**Table 3:** CZI OOB and correction factor in the turbid waters. ∆*λ* represents the shift of the effective central wavelength *λ_E_* relative to nominal one *λ_N_*.

		∆*λ* (nm)	OOB (Δ) *	OOB (%)	Correction Factor
Camera 1	B1	2.4(−2.5) ± 3.8	0.00003 (−0.00001) ± 0.00105	−0.13 ± 0.37	0.9823 ± 0.0729
	B2	−13.7(−1.5) ± 44.3	−0.00046 (−0.00007) ± 0.00231	−0.69 ± 0.33	1.0875 ± 0.0560
	B3	−15.5(−4.0) ± 49.0	0.00017 (0.00004) ± 0.00184	1.56 ± 1.45	0.9616 ± 0.0623
Camera 2	B1	3.1 (3.5) ± 4.0	0.00006 (−0.00001) ± 0.00145	−0.17 ± 0.46	0.9776 ± 0.0744
	B2	−13.7(−1.5) ± 44.3	−0.00068 (−0.00009) ± 0.00360	−0.68 ± 1.00	1.0985 ± 0.0618
	B3	−13.9(−2.5) ± 47.6	0.00019 (0.00004) ± 0.00195	2.06 ± 2.69	0.9671 ± 0.0993

* The numbers in the parentheses are the median.
